# Characterization of Melt-Spun Recycled PA 6 Polymer by Adding ZnO Nanoparticles during the Extrusion Process

**DOI:** 10.3390/polym16131883

**Published:** 2024-07-01

**Authors:** Anja Ludaš Dujmić, Rafaela Radičić, Sanja Ercegović Ražić, Ivan Karlo Cingesar, Martinia Glogar, Andrea Jurov, Nikša Krstulović

**Affiliations:** 1 Faculty of Textile Technology, University of Zagreb, Prilaz baruna Filipovića 28a, HR-10000 Zagreb, Croatia; anja.ludas@ttf.unizg.hr (A.L.D.); martinia.glogar@ttf.unizg.hr (M.G.); 2 Institute of Physics, Bijenička cesta 46, HR-10000 Zagreb, Croatia; rradicic@ifs.hr (R.R.); niksak@ifs.hr (N.K.); 3 Faculty of Chemical Engineering and Technology, University of Zagreb, Trg Marka Marulića 19, HR-10000 Zagreb, Croatia; icingesar@fkit.unizg.hr; 4 Department of Gaseous Electronics, Jožef Stefan Institute, Jamova Cesta 39, 1000 Ljubljana, Slovenia; andrea.jurov@ijs.si

**Keywords:** melt-spun recycled PA 6 polymer, ZnO nanoparticles, micromorphology, EDS chemical composition, FTIR analysis, thermal properties, mechanical properties

## Abstract

With recent technological advances and the growing interest in environmentally friendly fiber production processes, the textile industry is increasingly turning to the spinning of filaments from recycled raw materials in the melt spinning process as the simplest method of chemical spinning of fibers. Such processes are more efficient because the desired active particles are melt-spun together with the polymer. The study investigates the melt spinning of recycled polyamide 6 (PA 6) fibers modified with zinc oxide nanoparticles (ZnO NPs) in concentrations ranging from 0.1 to 2.0 wt% of the polymer. The extrusion process was optimized under laboratory conditions. An analysis of the effectiveness of the nanoparticle distribution and chemical composition was performed using scanning electron microscopy (SEM) with energy-dispersive X-ray spectroscopy (EDS), differential scanning calorimetry (DSC), and Fourier transform infrared spectroscopy (FTIR). The results of the thermal analysis show an increase in the glass transition temperature of the extruded material from 50.97 °C (raw polymer) to 51.40 °C to 57.98 °C (polymer modified with ZnO NPs) and an increase in the crystallization point from 148.19 °C to a temperature between 175.61 °C and 178.16 °C, while the molar enthalpy (ΔHm) shows a decreasing trend from 65.66 Jg^−1^ (raw polymer) to 48.23 Jg^−1^ (PA 6 2.0% ZnO). The FTIR spectra indicate PA 6 polymer, with a characteristic peak at the wavelength 1466 cm^−1^, but pure ZnO and PA 6 blended with ZnO show a characteristic peak at 2322 cm^−1^. The distribution of nanoparticles on the fiber surface is more or less randomly distributed and the different size of NPs is visible. These results are confirmed by the EDS results, which show that different concentrations of Zn are present. The mechanical stability of the extruded polymer modified with NPs is not affected by the addition of ZnO NPs, although the overall results of strength (2.56–3.22 cN/tex) and modulus of elasticity of the polymer (28.83–49.90 cN/tex) are lower as there is no drawing process at this stage of the experiment, which certainly helps to increase the final strength of the fibers. The results indicate the potential of modification with ZnO NPs for further advances in sustainable fiber production.

## 1. Introduction

At the beginning of the 20th century, several generations of synthetic fibers were developed. The fourth generation includes high-performance fibers for demanding applications such as microfibers and high-tech products with exceptional esthetics and comfort, as well as biodegradable fibers, nanofibers, and fibers with novel structures and properties. In response to strict environmental and economic considerations and the increasing global production of manmade fibers based on synthetic polymers, the focus is increasingly on recycled polymers and their use in fiber production [1,2].

Chemical melt spinning or extrusion is one of the most widely used methods for the production of synthetic fibers due to its cost efficiency, the absence of solvents, and the simplicity of the process. It is considered an environmentally friendly process compared to other technological processes. This method is used whenever a thermally stable melt with the required rheological properties can be produced from the polymer, which allows extrusion through a die without significant degradation of the polymer leading to the formation of filament fibers [3,4]. For polymers to be suitable for fiber production, they must meet requirements that enable chemical spinning and guarantee the required fiber properties while ensuring economically and ecologically advantageous production. The most commonly used polymers for melt spinning are polyamides, polyesters, and (linear) polyolefins [5,6].

Polyamide 6 (PA 6) is often used for fiber production due to its exceptional strength and wear resistance, making it ideal for products such as sports equipment and protective clothing. PA 6 is resistant to chemicals and temperatures and is therefore used in the automotive and chemical industries. Its low water absorption makes it suitable for outdoor use and its good mechanical properties enable a wide range of applications. PA 6 is also recyclable and, thus, contributes to sustainability and waste prevention. Due to its widespread use, PA 6 is used in the manufacture of various products for textile (e.g., clothing) and technical applications (e.g., fishing nets) [7,8,9,10,11,12].

Nanotechnology, which deals with the production of objects smaller than 100 nm, contributes to the development of materials with advanced properties for various applications. The production and use of synthetic fibers and additives contribute to the release of microplastics and other harmful pollutants into the environment and have a long-term negative impact on ecosystems. Therefore, the use of environmentally friendly production methods that involve a lower amount of additives is justified. Researchers are investing considerable efforts in the development of green synthesis methods for inorganic materials (e.g., metal nanoparticles) using microorganisms and plant extracts, which are more environmentally friendly, nontoxic, economical, and stable compared to other biological, chemical, and physical methods [13,14,15,16,17,18,19]. The textile industry is an important user of nanotechnology. Numerous nanotextiles on the market offer advanced properties and functions, such as self-cleaning, dirt and water repellency, oil resistance, antistatic properties, protection against UV radiation, antibacterial effect, and more. These nanotextiles belong to the category of functional high-performance materials [14,16,17,18].

Zinc oxide (ZnO) nanoparticles (NPs) are revolutionizing the textile industry by improving the functionality of textiles. These nanoparticles exhibit antibacterial properties that are ideal for maintaining hygiene in sportswear, underwear, and socks. In addition, ZnO NPs provide excellent UV protection and form an additional protective layer against harmful UV rays in outdoor clothing and sports equipment. Their antistatic properties are beneficial for workwear as they reduce electrical charges and minimize dust accumulation. In addition, these nanoparticles help deodorize textiles by absorbing unpleasant odors in items such as sportswear and footwear. Beyond functionality, the incorporation of ZnO NPs fulfills sustainability goals by providing improved textile properties without harming the environment [17,18,19,20].

In this work, the experiment focuses on the possibility of incorporating ZnO nanoparticles into the melt-spun filaments of the thermoplastic polymer PA 6. Following ecological principles, the research is carried out using recycled PA 6 polymer with post-consumer and preconsumer content (e.g., fishing net waste) as a starting material for extrusion and modification with defined nanoparticles. The integration of ZnO nanoparticles into a recycled PA 6 polymer melt is of particular interest for textile applications due to their antibacterial properties, their resistance to UV radiation, and their antistatic and odor-binding properties. The first part of the study focuses on analyzing the thermal stability of the polymer during extrusion, the yellowing index as a measure of polymer quality, the morphological properties of the surface, the mechanical properties of the unstretched filaments, and the chemical composition of the extruded/modified polymer filaments. The results obtained can help to draw conclusions about the efficiency of the polymer modifications obtained by using ZnO nanoparticles in a certain size range without deteriorating the properties of the material (polymer) for textile purposes.

## 2. Materials and Methods

Prior to extrusion, 600 g of PA 6 polymer (IUPAC name: poly(hexa-no-6-lactam), 2.4 relative viscosity, density 1.14 g/cm^3^, melt flow rate 59.1 cm^3^/10’, tensile strength at yield 78.48 MPa, and glass transition temperature for amorphous polymers of 226 °C [21], AquafilSLO d.o.o., Ljubljana, Slovenia) was dried at a temperature of 80 °C for 24 h. ZnO nanoparticles (Alfa Aesar GmbH & Co, Karlsruhe, Germany) with a size of 40 to 100 nm were added to the mass of PA 6 polymer in five different ratios (0.1%, 0.5%, 1.0%, 1.5%, and 2.0%).

The extrusion process was carried out using a Rondol Bench Top 21 mm twin-screw extruder (Rondol Technology Ltd., Staffordshire, UK). Before adding the dried polymer and the additive to the extruder feed, the parameters of the twin-screw extruder were optimized for the spinning process (listed in Table 1).

After the extrusion process, the samples were analyzed with a differential scanning calorimetry (DSC, Mettler Toledo d.o.o., Zagreb, Croatia) device, which measures the heat flow changes between the container with the sample and the reference container as a function of time and temperature under controlled pressure and inert atmosphere.

The second analysis was performed using a reflectance spectrophotometer (Data-color 850, Datacolor, Basel, Switzerland) to observe the change in yellowness index or whiteness of the polymer surface structure with increasing nanoparticle concentration. Scanning electron microscopy (SEM) and energy-dispersive X-ray spectroscopy (EDS) detector were performed using the Prisma E microscope (Thermo Fisher Scientific Inc., Waltham, MA, USA) to gain insight into the morphological and chemical properties of the samples. For the analysis of the samples with the scanning electron microscope, gold was vapor-deposited to obtain an electrically conductive sample on which the beams could be reflected. Using the EDS detector, a morphological view of the samples was created at a magnification of 800–1600× for the raw material PA 6. The other samples were imaged at a magnification of 8000×.

Fourier transform infrared spectroscopy (IR Spirit FTIR spectrophotometer, Shimadzu Europe GmbH, Duisburg, Germany) was performed to determine the ratio of the individual components in the sample as well as their quality and consistency. The samples were imaged with an FTIR spectrophotometer in the wavenumber range from 4000 to 500 cm^−1^.

The mechanical analysis was performed with the Strength Tester 3000 (MesdanLab, Brescia, Italy) using a tensile load cell of 100 daN, a distance between clamps of 50 mm, and testing speed of 100 mm/min at extension to break. Five measurements were taken per sample. Tenacity (cN/tex), work of rupture (cN·mm), and Young’s modulus (cN/tex) were calculated.

## 3. Results

**Figure 1:**
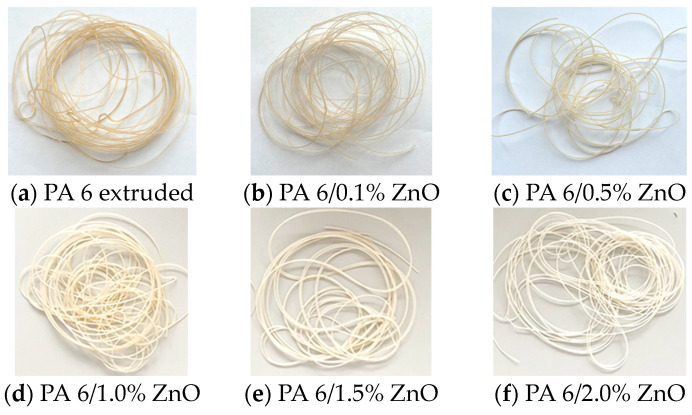
Melt-spun filaments of extruded and modified polymer with adding of ZnO nanoparticles (**a**–**f**).

After optimizing the process parameters of melt-spun filaments depending on the thermal properties of the PA 6 polymer with the addition of various concentrations of ZnO nanoparticles, the modified filament shown in Figure 1 was obtained.

### 3.1. Differential Scanning Calorimetry

DSC is a technique that continuously monitors the difference in heat flow between the sample and the reference material as a function of temperature or time. Instead of the usual linear temperature program, a sinusoidal (modulated) heating rate is used. The temperature range of the device extends from −150 °C to 700 °C. Table 2 gives an overview of the results obtained with the DSC device. All samples were heated and cooled in two cycles to quench their thermal history in the first cycle.

The glass transition point (Tg) is the temperature range expressed by the mean value of the temperature transition from the glassy to the rubbery state and vice versa. The glass transition point was successfully measured using the differential scanning calorimetry method. The values show no trend, except that the glass transition point values for the extruded material are higher than for the raw material (50.97 °C), with temperatures ranging from 51.40 °C to 57.98 °C.

Crystallization (Tc) is the process by which the particles of a substance arrange themselves in regular, repeating patterns called crystals. All samples, with the exception of the raw material PA 6, behave similarly. The raw polymer PA 6 shows a change during the heat treatment, and the value of the indicated temperature moves from 148.19 °C to a temperature between 175.61 and 178.16 °C. All values of the extruded material are higher than those of the raw material, as is the glass transition point.

**Figure 2:**
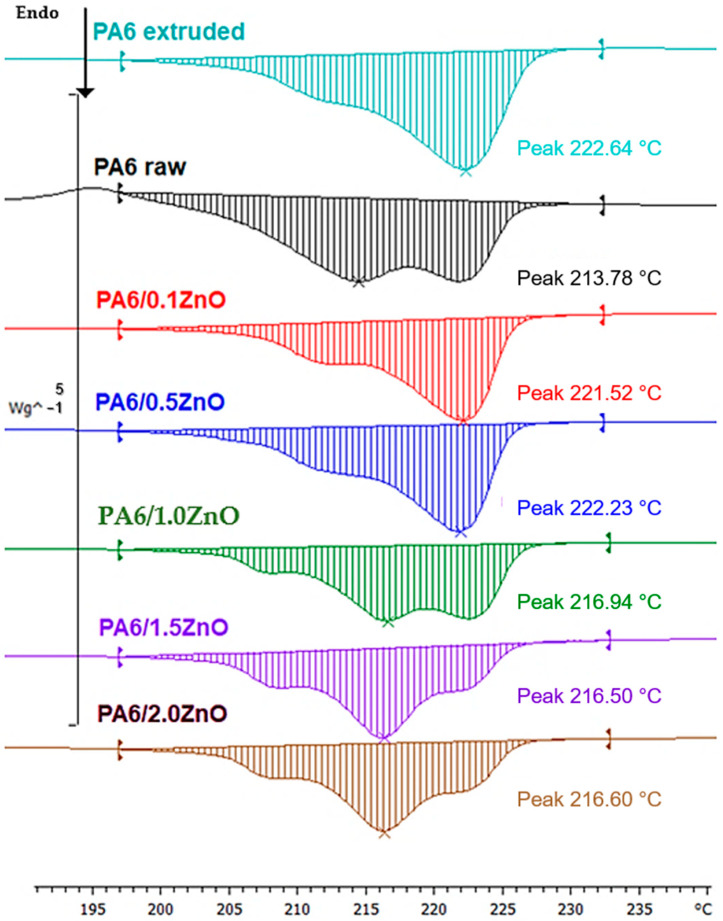
Molar enthalpy and maximum melting temperature, determined from the second heating cycle.

The melting point (Tm) is the temperature range at which a substance changes from a solid to a liquid state. Just as with the glass and crystallization points, all values have a melting point peak that is shifted towards higher temperatures. It is interesting to note that all samples, including the raw material, exhibit polymorphism. In this case, there are two different crystal lattices in which the material crystallized: hexagonal (melting point around 215 °C) and monoclinic (melting point from 220 °C to 230 °C). The molar enthalpy (ΔHm) shows a decreasing trend, with the exception of the PA 6/0.5% ZnO sample. The maximum melting temperature values listed in Table 2 and Figure 2 indicate the temperature at which the melting rate is highest.

### 3.2. Spectrophotometric Analysis

Based on the spectral characteristics of seven samples measured with the reflectance spectrophotometer according to DIN 6167, the degree of whiteness according to the CIE system and the yellowness index were calculated [22]. Table 3 shows the results of the degree of whiteness effect and the yellowing index.

The presented results show that the initial polymer sample (PA 6 raw) has a higher degree of whiteness and a lower yellowing index. Comparing the other results with the first extruded sample without ZnO nanoparticles addition (PA 6 extruded), the results are in line with expectations considering the visual appearance of the samples. The sample with the addition of 2.0% ZnO achieves the highest purity of whiteness and has the lowest yellowing index compared to samples with PA 6 0.1 to 1.5%.

**Figure 3:**
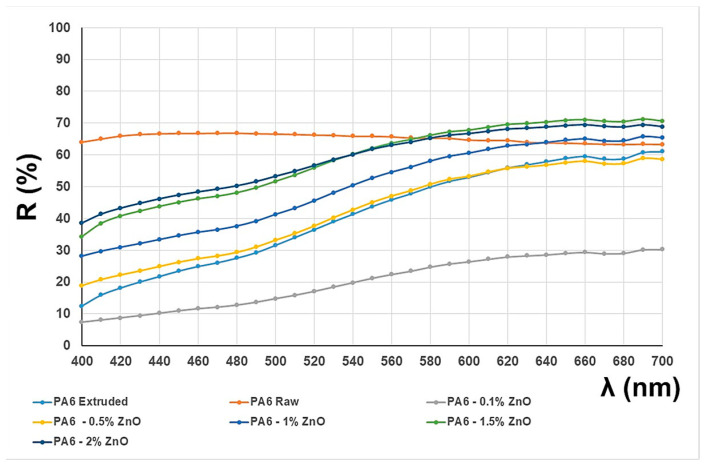
Reflectance curves.

The reflectance curves obtained by spectrophotometric measurement (Figure 3) are characteristic of achromatic colors. In achromatic colors, there is a relatively uniform reflection of all parts of the spectrum without accentuated peaks that would indicate the wavelengths of the predominant absorption. However, the colors, although achromatic, show a slight yellowing, which is confirmed not only by the value of the yellowing index, but also by the slope of the curve. It shows a slightly lower reflectance in the blue–violet and blue areas, which is consistent with the yellowing results, and it is obvious that the samples are not white but yellowish. Only the reflectance curve obtained for the raw PA 6 sample shows a characteristic course for white color—a uniform reflection of all parts of the spectrum at a high percentage and a straight course of the curve without characteristic slopes. Also, a slight increase in the reflectance curve for the raw PA 6 sample in the blue–violet waveband indicates the slight presence of additives that can be associated with florescence and optical whiteness.

### 3.3. Morphological and Chemical Analysis Using Scanning Electron Microscopy with Energy-Dispersive X-ray Spectroscopy

**Figure 4:**
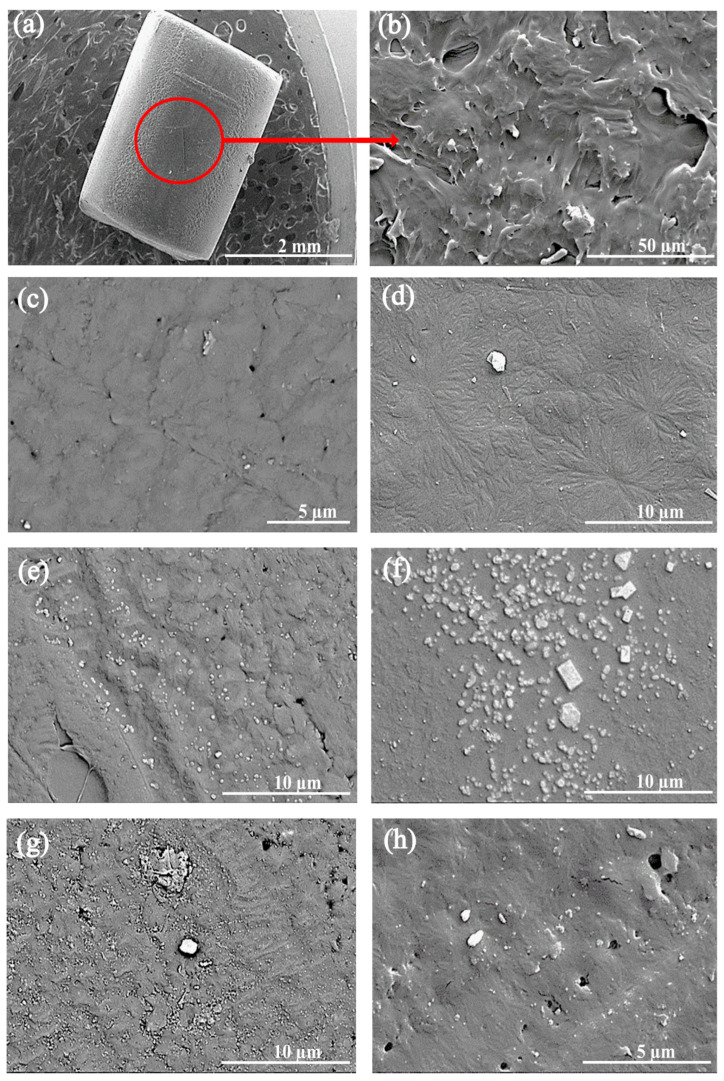
Morphological SEM analysis of PA 6 and extruded PA 6 polymers with different concentrations of ZnO nanoparticles: (**a**,**b**) sample of PA 6 granulate with magnifications of 800–1600×; (**c**–**h**) sample of PA 6 filaments with magnifications up to 8000×.

The morphological SEM analysis shows that the ZnO nanoparticles on the surface of the filaments have white, irregular shapes compared to the reference sample of PA 6 polymer. When the concentration of the nanoparticles is increased, an increase in the white particles on the surface of the polymer can be observed. It is noticeable that the surface of the filaments acquires an increasingly pronounced relief structure with surface roughness as the ZnO concentration increases (Figure 4). A random distribution of nanoparticles on the polymer surfaces is observed, with agglomerates forming at higher concentrations of nanoparticles.

**Figure 5:**
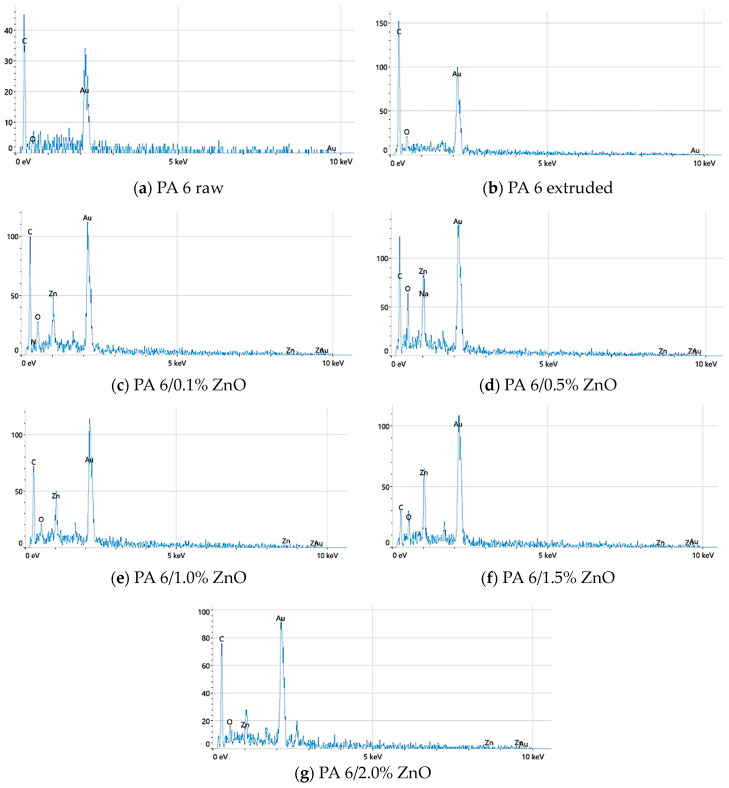
Energy-dispersive X-ray spectroscopy.

Energy-dispersive X-ray spectroscopy is an analytical technique for determining the chemical composition of the sample under investigation. Figure 5 shows the elements that were detected with the highest percentages in the EDS analysis: gold (Au), zinc (Zn), carbon (C) and oxygen (O). The presence of gold is attributed to the fact that the samples were previously coated with gold in order to achieve the electrical conductivity for the scanning electron microscope analysis. The presence and amount of zinc is obviously due to the addition of ZnO nanoparticles, which increased more or less proportionally with the increase in their concentration in the PA 6 polymer. Therefore, we can confirm that even small amounts (0.1%) of ZnO nanoparticles were incorporated into the PA 6 polymer. The presence of sodium (Na) in Figure 5d belongs to the impurities during the examination and can be neglected. The above microscopic images (Figure 4c–h) show a random distribution of particles on the surface of the polymer matrix and suggest a similar distribution inside the polymer. Such results may be the result of a limited surface area scanning the micromorphological surface, especially at higher magnifications, which is also confirmed by the EDS data in Figure 5c–g.

### 3.4. Infrared Spectroscopy with Fourier Transmission

**Figure 6:**
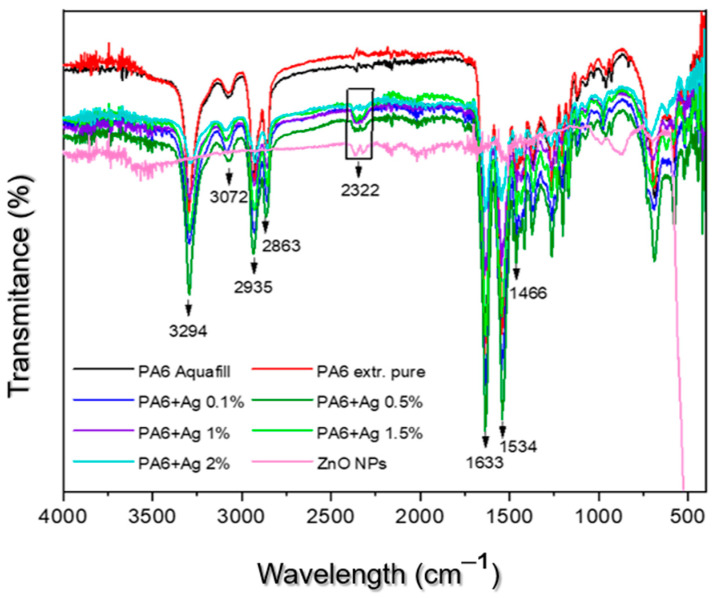
FTIR spectrum of modified polymers.

In Figure 6, the most important transmission bands (peaks) correspond to the functional groups of the PA 6 polymer. In the fingerprint of the PA 6 polymer, the following characteristic peaks at 3294 cm^−1^ can be seen, which correspond to the stretching of the –NH group. The presence of characteristic peaks at 3072 cm^−1^ is due to the presence of the amide II (C-N) group, while 2935 cm^−1^ and 2863 cm^−1^ correspond to the asymmetric and symmetric stretching of the CH_2_ groups and 1633 cm^−1^ corresponds to the stretching of the amide I (C=O) group. Characteristic peaks at 1534 cm^−1^ correspond to the bending of the –NH group, at 1466 cm^−1^ to the specific absorption of PA 6, and at 2322 cm^−1^ to the vibration of the O=C=O groups.

A review of the relevant literature led to the following results. The FTIR spectra indicate that it is a polyamide polymer, with a characteristic peak at the wavelength of 1466 cm^−1^. The absence of the characteristic ZnO peak at 500 cm^−1^ could be due to the low ZnO content in the polymer. However, pure ZnO and PA 6 blended with ZnO show a characteristic peak at 2322 cm^−1^, which is probably due to the vibration of CO_2_ under the influence of moisture. It is known that ZnO can absorb CO_2_ at the surface. Therefore, we can assume that the peak in PA 6 with the addition of zinc oxide is due to CO_2_ absorption, which was not observed in the raw PA 6 sample [23].

### 3.5. Mechanical Analysis

Table 4 shows the results of the mechanical properties of melt-spun PA 6 filaments by calculating the strength (cN/tex), work of rupture (cN·mm), and Young’s modulus of elasticity (cN/tex) for polymer filaments before and after modification by the addition of ZnO NPs.

The present results (Table 4) show lower values (2.56–3.22 cN/tex) for the tenacity and elastic modulus of the polymer (28.83–49.90 cN/tex) compared to the results from the literature. The tenacity of PA 6 is 30–40 cN/tex, the elongation at break is 20–45%, and the initial modulus for the standard fiber type is 50–300 cN/tex [7], since the polymer had not yet been subjected to the stretching process to achieve the desired fineness and tensile strength. The work of breakage provides information about the elongation of the yarn after reaching the maximum force at which breakage occurs.

## 4. Discussion

According to the results presented, this study characterized the effects of the incorporation of ZnO NPs on the thermal properties of PA 6 by using differential scanning calorimetry to analyze the changes in glass transition temperature, crystallization temperature and melting point. The glass transition temperature increased from 50.97 °C in the raw polymer to a range of 51.40 °C to 57.98 °C in the modified polymers, indicating improved thermal stability and increased chain rigidity due to the interaction between the ZnO NPs and the polymer matrix. This rigidity can improve the performance of the material under thermal stress. The crystallization temperature increased from 148.19 °C in the raw PA 6 to a range between 175.61 °C and 178.16 °C in the samples modified with ZnO NPs. This shift indicates that the ZnO NPs act as potential nucleating agents, facilitating crystallization at higher temperatures and possibly leading to a more ordered crystalline structure. The difference between the melting temperatures of raw PA 6 and extruded PA 6 is shown by a slight increase in melting point from 213.78 °C to 222.64 °C, indicating that a more oriented structure is formed during the extrusion process compared to the raw amorphous polymer. During modification with ZnO NPs, there is a slight decrease in melting point (compared to the extruded PA 6 sample) and the presence of polymorphism, with PA 6 crystallizing in both hexagonal and monoclinic forms [7]. The molar enthalpy (ΔHm) shows a decreasing trend from 65.66 J/g in the raw polymer to 48.23 J/g in the PA 6/2.0% ZnO sample, indicating a decrease in crystallinity with increasing ZnO concentration. This decrease could be due to the interaction of the ZnO NPs with the polymer chain, which could hinder its ability to organize into crystalline structures.

The tested PA 6 raw sample initially showed a high degree of whiteness and a low yellowing index, which is typical for untreated polymer. In general, the extrusion process can lead to increased yellowing of the polymer, for which there are several reasons: Thermal degradation due to higher temperatures during extrusion can cleave the polymer chains and form colored degradation products; exposure to oxygen at high temperatures can lead to oxidative degradation products that cause discoloration; impurities such as residues of catalysts or additives that can react at high temperatures and form colored compounds; shear stresses during extrusion can cause chemical changes and form colored compounds, etc. The presence of yellowish colors after extrusion of the PA 6 filaments could be caused by residues in the extrusion system (even if it was cleaned before the process) or by possible residues in the recycled polymer granules from the manufacturer. The addition of ZnO NPs, especially at higher concentrations (up to 2.0%), improved the whiteness and minimized the yellowing index. Detailed explanations can be found in Section 3.2.

As described in Section 3.3, the results of scanning electron microscopy and energy-dispersive X-ray spectroscopy provide detailed insights into the distribution and micromorphology of the ZnO NPs on the fibers. The SEM images show a random distribution of particles on the surface of the polymer matrix and indicate a similar distribution inside the polymer, with pronounced surface roughness and agglomeration increasing at higher NP concentrations. EDS analysis confirms the presence of Zn with concentrations consistent with the added ZnO, indicating effective incorporation and distribution of the nanoparticles in the polymer.

Fourier transform infrared spectroscopy confirms the incorporation of ZnO NPs into the PA 6 matrix. Characteristic peaks of PA 6 are observed, together with a peak at 2322 cm^−1^ in the ZnO-modified samples, which probably correlates with CO_2_ absorption by ZnO [23]. This suggests that the ZnO retains its functional properties within the polymer matrix and may provide additional benefits such as antimicrobial activity, UV resistance, and other functionalities.

The mechanical tests show that the addition of ZnO NPs does not affect the mechanical stability of the PA 6 fibers. However, the strength and modulus of elasticity are below the values usually reported for PA 6 fibers, which is primarily due to the fact that no stretching process was performed in this study. Drawing is a crucial step that aligns the polymer chains and improves the mechanical properties. Although the mechanical properties are below the standard value for fully processed PA 6 fibers, they indicate that further optimization, especially through the drawing process, could improve the mechanical properties, making these fibers suitable for various textile applications.

## 5. Conclusions

With growing interest in environmentally friendly fiber production processes, the textile industry is increasingly turning to the spinning of filaments from recycled raw materials in the melt spinning process as the simplest method of chemical spinning of fibers.

Such processes are more efficient because the desired active particles are melt-spun together with the polymer, while the added active particles are less firmly bound to the fiber/textile during subsequent treatments and are, therefore, easier to remove during textile care and wear.

The results of this study underline the potential of ZnO-modified PA 6 fibers to promote sustainable textile production. The observed changes in thermal properties combined with the obtained chemical functionality and promising mechanical properties indicate that these fibers can be effectively used in a wide range of applications.

The observed positive changes in thermal stability and crystallization behavior of PA 6 fibers indicate that ZnO NPs can improve the performance of recycled polymers, which is beneficial for applications requiring higher heat resistance, such as automotive textiles, industrial textiles, and certain types of functional clothing.

From a chemical point of view, the successful integration of ZnO NPs into the PA 6 matrix, as confirmed by FTIR and EDS analyzes, opens up the possibility of producing fibers with additional functionalities such as UV protection, antibacterial properties, and improved durability.

The mechanical properties of the ZnO-modified PA 6 fibers are lower than those reported in the literature for fully processed PA 6. They show potential for improvement through further processing drawing and annealing so that the mechanical performance of these fibers can be improved to meet industry standards. Future research should focus on optimizing the drawing process to improve the mechanical properties and on exploring practical applications in different textile and industrial contexts. In addition, investigating the long-term stability and functional performance of these fibers under real-life conditions is crucial for their successful introduction into sustainable fiber production.

Furthermore, the sustainable aspect of using recycled PA 6 in combination with the additional functionality of ZnO NPs meets the growing demand for environmentally friendly materials in the textile industry. By using recycled materials, the environmental impact of textile production is reduced, contributing to more sustainable manufacturing practices.

## Figures and Tables

**Table 1:** Optimization of a twin-screw extruder for the melt spinning of PA 6 polymer.

Pressure	Torque	Screw Diameter	Six Heating Zones
0.7–2.0 bar	50–80 rpm	21 mm	1st zone: 220 °C, 2nd to 5th zones: 230 °C 6th zones: 233 °C

**Table 2:** Results of polymer thermal properties by differential scanning calorimetry.

Samples	Tg (°C)	Tm (°C)	ΔHm (J g^−1^)	Tc (°C)
PA 6 raw	50.97	213.78	65.66	148.19
PA 6 extruded	51.40	222.64	63.93	175.61
PA 6/0.1% ZnO	57.50	221.52	50.19	176.48
PA 6/0.5% ZnO	52.56	222.23	53.66	175.88
PA 6/1.0% ZnO	57.98	216.94	49.50	176.92
PA 6/1.5% ZnO	54.27	216.50	47,90	178.16
PA 6/2.0% ZnO	51.55	216.60	48.23	177.59

**Table 3:** Results of the whiteness and yellowing index.

Samples	Whiteness CIE	Yellowing Index
PA 6 raw	1.89	−2.43
PA 6 extruded	−29.54	62.65
PA 6/0.1% ZnO	−34.28	66.10
PA 6/0.5% ZnO	−26.37	55.86
PA 6/1.0% ZnO	−20.99	45.71
PA 6/1.5% ZnO	−13.37	34.67
PA 6/2.0% ZnO	−11.00	29.44

**Table 4:** Results of tensile strength of undrawn PA 6 filaments.

Samples	Tenacity (cN/tex)	CV (%)	Work (cN·mm)	CV (%)	Young Modulus (cN/tex)
PA 6 extruded	2.78	36.6	6383	52.6	37.98
PA 6/0.1% ZnO	3.16	18.9	33,861	31.9	45.66
PA 6/0.5% ZnO	2.56	17.9	29,617	20.5	39.09
PA 6/1.0% ZnO	2.90	9.3	29,999	7.4	28.83
PA 6/1.5% ZnO	3.22	26.6	24,208	24.9	49.90
PA 6/2.0% ZnO	2.84	18.7	13,910	16.1	35.73

## Data Availability

Data are contained within the article.
